# Vaccination with the Inactivated Vaccine (Sinopharm BBIBP-CorV) Ensures Protection against SARS-CoV-2 Related Disease

**DOI:** 10.3390/vaccines10060920

**Published:** 2022-06-09

**Authors:** Chao Wang, Lin-Yi Chen, Qing-Bin Lu, Fuqiang Cui

**Affiliations:** 1 Department of Laboratorial Science and Technology & Vaccine Research Center, School of Public Health, Peking University, Beijing 100191, China; wchao@bjmu.edu.cn (C.W.); chenlinyi137@pku.edu.cn (L.-Y.C.); 2 Global Center for Infectious Disease and Policy Research & Global Health and Infectious Diseases Group, Peking University, Beijing 100191, China

**Keywords:** inactivated SARS-CoV-2 vaccine, COVID-19, BBIBP-CorV

## Abstract

Vaccination against coronavirus disease 2019 (COVID-19) has become an important public health solution. Developing a safe and effective vaccine against COVID-19 is a viable long-term solution to control the pandemic. As one of the two inactivated severe acute respiratory syndrome virus 2 (SARS-CoV-2) vaccines developed in China that entered the WHO emergency use list, Sinopharm BBIBP-CorV, an aluminum-hydroxide-adjuvanted, inactivated whole-virus vaccine, has been widely distributed, with more than 400 million doses administered in more than 40 countries. The evidence of the safety, efficacy, and effectiveness of BBIBP-CorV is gathered and reviewed. We further comment on one of the latest papers that disclosed the effectiveness results between BBIBP-CorV, rAd26-rAd5, and ChAdOx1.

## 1. Introduction

The coronavirus disease 2019 (COVID-19) has caused huge social, economic, and health losses worldwide. By 18 May 2022, more than 519 million COVID-19 cases and 6 million deaths had been confirmed [1]. Vaccines for severe acute respiratory syndrome coronavirus 2 (SARS-CoV-2) have been claimed as necessary to combat the tragic health and socioeconomic consequences of the pandemic. Various strategies have been used for the development of vaccines for SARS-CoV-2, among which the inactivated virus platform is one of the most mature and traditional techniques in designing effective and safe vaccines against SARS-CoV-2 [1]. As of 3 November 2021, the WHO has approved eight SARS-CoV-2 vaccines for emergency use [2]. National regulators have also assessed other SARS-CoV-2 vaccine products for emergency use in their countries. In the meantime, 130 SARS-CoV-2 vaccines are under clinical development, and 194 SARS-CoV-2 vaccines are in the pre-clinical development stage [3].

As one of the two inactivated SARS-CoV-2 vaccines developed in China that was included in the WHO emergency use list, Sinopharm BBIBP-CorV, an aluminum-hydroxide-adjuvanted, inactivated whole-virus vaccine, has been widely distributed, with more than 400 million doses in more than 40 countries [1]. The virus strain used to produce SARS-CoV-2 vaccine BIBP was derived from clinical isolates from Chinese CDC in January 2020, which is inoculated on Vero cells for culturing, virus harvesting, β-propiolactone inactivation, concentration, purification, ultrafiltration, and sterile filtration. Adsorption on aluminum adjuvant takes place during final bulk formulation. Till now, variants of concern have frequently made effective changes in the virus’s “Spike” protein. The inactivated vaccine contains the whole virus component, which ensures the vaccine’s efficacy in terms of an increasingly complicated scenario with the surge of virus variants due to mutations associated with increased viral transmission and, in some cases, neutralizing antibody escape. However, the evidence for its actual efficacy is heterogeneous and has been demonstrated across the world. We searched references from the mainstream database, including PubMed, Medline, and Research gate. The searching strategy is set as (((“efficacy” [Title/Abstract] OR “effectiveness” [Title/Abstract] OR “Immunogenicity” [Title/Abstract] OR “safety” [Title/Abstract]) OR “side effect” [Title/Abstract] OR “Neutralizing activity” [Title/Abstract]) AND ((COVID-19 [Title/Abstract]) OR (SARS-CoV-2 [Title/Abstract])))) AND (((“inactivated vaccine” [Title/Abstract]) OR (“BBIBP-CorV” [Title/Abstract])) OR (Sinopharm [Title/Abstract]))). The original literatures and reports were screened and recruited, and reviews were excluded for brevity. As it is claimed that more vaccines should be distributed among the wider public, an interim effectiveness and safety assessment is needed for this widespread inactivated vaccine before its further vaccination [2].

## 2. Safety of the Inactivated SARS-CoV-2 Vaccine

Safety of Sinopharm BBIBP-CorV has been identified from unsolicited surveillance data pre and post authorization from China, UAE, and Bahrain in adults aged 18 to 59 [2,4,5]. In adults aged ≥60 years, the safety profile was comparable to that in younger adults, but they presented a lower reactogenicity [2]. As of 8 March 2021, 35.4 million people had received at least one dose of Sinopharm BBIBP-CorV in China; by then, there were 6918 reports of adverse reactions, including 4633 general adverse reactions [2]. The overall incidence of adverse reactions was 19.6/100,000 doses, and the incidence of general reactions was 13.1/100,000 doses. Interim reports have shown the point estimates of vaccine efficacy between 64% and 100%, with the seroconversion of neutralizing antibodies of 99.5% and 100%, in the groups of the 18–59 years and ≥60 years, respectively [2,6]. Additionally, trials in children were completed for BBIBP-CorV between 14 August 2020 and 24 September 2020, which were safe and well-tolerated, and showed robust humoral responses against SARS-CoV-2 infection after two doses’ injection [7]. A third boosting dose has been shown to be safe and could reduce the Omicron escape from neutralization by homologous boosters or heterologous boosters such as the protein subunit vaccine (e.g., ZF2001, BNT162b2) [8,9,10]. Based on the data of 5.9 million people vaccinated with Sinopharm BBIBP-CorV in China, 1453 people reported adverse events for a reporting rate of 24.6/100,000 doses. Of the 108 local reactions reported, there were two reports of severe induration and six reports of severe redness and swelling. Of 202 cases of fever reported, 86 were classified as severe (≥38.6 °C). All 11 cases with facial nerve symptoms were assessed to be unrelated to the vaccine. Reports also suggested the good safety of BBIBP-CorV after the boosting dose from China and Iran in the real-environment [11,12,13,14]. Adverse events among individuals who received BBIBP were suggested significantly less frequently than the other vaccine groups [15]. These data further confirmed the vaccine safety in the real-environment deployment [16].

## 3. Efficacy of Sinopharm BBIBP-CorV

Clinical trials conducted during the first wave of COVID-19 suggested BBIBP-CorV offered satisfactory efficacy in preventing new infections and death related to SARS-CoV-2. The estimated protective efficacy was 78.89 % (95% CI 65.79%, 86.97%). Vaccine efficacy, calculated by taking into consideration the person-years of follow-up, was 78.07% (95% CI 64.82%, 86.33%). Vaccine efficacy estimates were similar in males and females (point estimates of 78.4% and 75.6%, respectively) [2]. With respect to the kinetics and persistence of immune responses, the studies indicated that the T cell response persisted while the antibody response declined three months following BBIBP-CorV [17,18]. In regard to SARS-CoV-2 variants, observation trails showed that neutralizing sensitivity and immunogenicity dropped nearly 60% against the prototype, Beta, and Delta, while it might have escaped the Omicron variant 180 days after the second dose [19]. The study conducted in Sri Lanka also showed that the total antibody levels and ACE2R-Ab declined 2 to 12 weeks after the second dose, while ex vivo T cell responses remained unchanged. The decline in ACE2R-Ab levels was significant among older age groups [17]. Since BBIBP-CorV has been reported with a lower anti-spike IgG, a heterologous booster that inspires a higher neutralizing antibody might be a good solution to prevent the variants of concern (VOC) breakthrough infection such as the Omicron variant [10].

## 4. Effectiveness within the Real-Environment

The RCTs of Sinopharm BBIBP-CorV were conducted under the restrict protocol at a time when the VOC had not yet been prevalent (Table 1). The safety and effectiveness of BBIBP-CorV is therefore needed for evaluation after engaging in marketing in the real world. Regarding the effectiveness of the Sinopharm BBIBP-CorV, the WHO reported a test-negative design performed in Bahrain in which the vaccine effectiveness was around 90% for adults by primary analysis based on 14 days post the second dose [16]. By AlHosani’s evaluation in Abu Dhabi Emirate, the United Arab Emirates (UAE), the levels of effectiveness of the BBIBP-CorV in fully vaccinated individuals against hospitalization, severe diseases, and death were 80%, 92%, and 97%, respectively. Furthermore, a single-dose vaccination was much less effective in preventing hospitalization, severe diseases, or death. As is known, the first dose only triggers the immune system, while the second dose is needed to elicit the dominant immune response [20]. A study conducted in Serbia has confirmed that specific antibodies against SARS-CoV-2 were detected at a low level at 1.5 to 3 months post the first dose of vaccination of BBIBP-CorV [21]. The finding suggests that a second dose is critical for developing an effective immune response, and further boosters may be needed to increase and retain vaccine effectiveness. Another study conducted in Peru indicated a similar effectiveness in preventing all-cause or COVID-19-specified mortality (90.5% and 93.9%, respectively). However, its effectiveness in preventing SARS-CoV-2 infection was reported as 49.2% to 54.7% for all the participants and people aged ≥ 60 years, respectively [22].

The study conducted in Guangzhou city of China similarly indicated that the vaccine effectiveness for the two-dose vaccination was estimated to be 59.0% (95% CI 16.0% to 81.6%) against SARS-CoV-2, and 70.2% (95% CI 29.6% to 89.3%) against moderate illness caused by the Delta variant, even though it did not report stratified effectiveness between BBIBP-CorV and CoronaVac [23], which was reported in Zhengzhou city of China [24]. One study conducted in the United Arab Emirates also indicated that the inactivated vaccine BBIBP-CorV and the mRNA vaccine BNT162b2 demonstrated protection against COVID-19-related hospitalizations caused by the Delta variant with 95% (95% CI 94% to 97%) and 98% (95% CI 86% to 99%) effectiveness, respectively [25].

In the city of Buenos Aires, Argentina, parallel comparisons were reported among various SARS-CoV-2 vaccines, including rAd26-rAd5, ChAdOx1, and BBIBP-CorV. Vaccination was associated with an 88.1% (95% CI 86.8% to 89.2%) decrease in incidence for those who received the full vaccination schedule (two doses) and a 47.2% (95% CI 44.2% to 50.1%) reduction for those who received only one dose. The two-dose schedule was associated with a 94.0% (95% CI 93.0% to 94.8%) reduction in incidence for people aged 70 to 79 years. For persons aged at least 80 years, the effectiveness was reported as 88.4% (95% CI 86.9% to 89.8%), cumulatively. A single dose was associated with a 65.8% (95% CI 61.7% to 69.5%) reduction in mortality, and the full vaccination schedule of two doses was associated with a 96.6% (95% CI 95.3% to 97.5%) reduction, cumulatively. The study did not report separated effectiveness for the three vaccines but represented a hazard ratio. Based on the data reported in the paper, we further calculated and assessed the specified effectiveness of the three vaccines. As estimated, the incidence among the vaccinated population of BBIBP-CorV, rAd26-rAd5, and ChAdOx1 was around 5.1 per 100,000 person-years; 3.1 per 100,000 person-years; and 3.0 per 100,000 person-years, respectively, with a corresponding vaccine effectiveness of 86%, 93%, and 93%, respectively. The all-cause mortality was estimated as 0.50 per 100,000 person-years for BBIBP-CorV; 0.17 per 100,000 person-years for rAd26-rAd5; or 0.25 per 100,000 person-years for ChAdOx1, with a corresponding effectiveness of 79%, 92%, and 90%, respectively. However, it is confusing that there is a big gap between the mixed effectiveness (96.6%, 95% CI 95.3% to 97.5%) and the separated effectiveness (79%, 92%, and 90%) in preventing all-cause mortality. The separated vaccine effectiveness to mortality reduction was also much lower than that to the incidence reduction, as (79%, 92%, and 90%) vs. (86%, 93%, and 93%), which is contradicted by the conclusions of other similar studies [20,21,22,23,26]. In any case, such results still indicated that vaccination against SARS-CoV-2 has comparable effectiveness at protecting people from disease or death if they are given a full vaccine schedule [27].

## 5. Conclusions

This summary has the drawback that we could not cope with the various definitions, including “vaccine efficacy”, forcibly caused by the broad inclusion of related studies. Nevertheless, the Sinopharm BBIBP-CorV was included in the first wave of emergency use due to its safety and efficacy, which was guaranteed by the inactivated vaccine technique. Compared to vaccines on other platforms, such as mRNA vaccines, BBIBP-CorV shows less immunogenicity and durability in laboratory tests. Despite its lower level of efficacy and immunology, its real-world effectiveness in preventing people from severe diseases except infection still proved to be valuable. Full vaccination with two doses is the basic requirement for the protective effect. A heterogeneous boosting dose of BBIBP might be an effective solution to reduced vaccine efficacy against emerging VOCs in the future, while the most urgent matter at hand is to promote the deployment and equity of vaccination around the world, whatever the kind of vaccine that is accessible.

## Figures and Tables

**Table 1:** A summary of the safety, immunogenicity, and effectiveness of BBIBP-CorV.

Author	Year	Country	Study Design	Age Range	Immunogenicity (GMT (95% CI))/Effectiveness (PE/PR (95% CI))	General Reaction	Severe Reaction
BIBP [2]	2021	China	RCT phase 3	18–59 years	NAbs:152.6 (146.0~159.4)	20.45%	None
				≥60 years	NAbs:109.7 (97.4~123.4)	NA	NA
Xia [4]	2020	China	RCT phase 1	18–59 years	42 days NAbs: 2 μg: 87.7 (64.9~118.6) 4 μg: 228.7 (186.1~281.1) 8 μg: 211.2 (158.9~280.6)	34/72	None
				≥60 years	42 days NAbs: 2 μg: 80.7 (65.4~99.6) 4 μg: 131.5 (108.2~159.7) 8 μg: 170.9 (133.0~219.5)	14/72	None
			RCT phase 2	≥18 years	28 days NAbs: 4 μg:218·0 (181.8~261.3)	78/336	None
Xia [6]	2022	China	RCT phase 1/2	3–5 years	28 days NAbs: 2 μg: 14.5 (95% CI 11.2~18.7) 4 μg: 20.2 (16.3~25.0) 8 μg: 21.3 (17.5~26.0)	78/251	None
				6–12 years	28 days NAbs: 2 μg: 30.0 (25.0~35.9) 4 μg: 48.0 (42.5~54.3) 8 μg: 54.1 (45.6~64.1)	65/252	None
				13–17 years	28 days NAbs: 2 μg: 61.4 (52.1~72.3) 4 μg: 55.8 (45.3~68.7) 8 μg: 80.2 (69.3~92.9)	86/252	None
Elgendy [5]	2022	Egypt	Online Survey	≥18 years	3 weeks post 2nd dose IgG anti-spike-protein antibodies: 40 AU/mL	50%	8%
Moghnieh [10]	2021	Lebanon	Cohort: boosting and heterologous dose of BBIBP	≥18 years	3 months post 2nd dose BBIBP NAbs: 9 (6~13) BNT162b2 booster dose: 8040 (4612~14,016)	31/50	None
Ariamanesh [14]	2021	Iran	Post-Market study	Malignancy patients	NA	0.7%~15.4%	0.4%~2.2%
Zhang [11]	2022	China	Open-label trial: homologous booster of BBIBP	≥18 years	14 days post 3rd dose NAbs: 556.9 (494.9~619.0) 28 days post 3rd dose NAbs: 447.3 (401.7~492.9)	No significant	None
Yu [19]	2022	China	Observational clinical trial	18–59 years	21 days after the first dose NAbs: 5.32 (2.33~13.35) 28 days after the 2nd dose NAbs: 33.96 (12.56~82.04) NAbs remained in 43.64% of participants for Delta 180 days afterwards:15.93 (12.72~19.94)	44.43% (447/1006)	None
Assawakosri [15]	2022	Thailand	Prospective cohort study	18–70 years	Heterologous booster of BBIBP 180 days after 2nd dose: 1294.54 (1084.08~1545.87)	42.1%	None
Ismail [20]	2021	UAE	Retrospective cohort study	≥15 years	Hospitalization 79.8% (78~81.4%) Critical care 92.2% (89.7~94.1%) Death 97.1% (83~99.9%)	NA	NA
Javier [22]	2021	Peru	Retrospective cohort study	18–100 years	Infection 40.3% (38.9~41.6%) All-cause mortality 83.6% (80.2~86.4%) COVID-19 mortality 88.7% (85.1~91.4%)	NA	NA
Li [23]	2021	China	Test-negative case–control study	18–59 years	Delta variant Infection 59.0% (16.0~81.6%) Moderate illness 70.2% (29.6~89.3%) Death 100% against Delta variant	NA	NA
Wu [24]	2021	China	Retrospective cohort study	≥18 years	Delta variant Symptomatic disease 50.56% (3.8~74.6%) Pneumonia 54.71% (−3.4~80.2%) Severe case 82.41% (21.0~96.1%)	NA	NA
Mousa [25]	2022	UAE	Not reported	≥18 years	Delta variant; hospital admission 95% (94~97%)	NA	NA
Rearte [26]	2022	Argentina	Test-negative case–control study	≥60 years	COVID-19 mortality 85.0% (84.0~86.0%)	NA	NA
Macchia [27]	2021	Argentina	Cohort study	≥60 years	Infection 86.0%; all-cause mortality 79%	NA	NA

Note: NAbs, neutralizing antibody; GMT, geometric mean titer; PE, protective efficacy; PR, protection rate; and COVID-19, coronavirus disease 2019.

## Data Availability

Not applicable.

## References

[1] World Health Organization WHO Coronavirus (COVID-19) Dashboard. https://covid19.who.int/.

[2] World Health Organization (2021). WHO Recommendation COVID-19 Vaccine BIBP/Sinopharm. https://extranet.who.int/pqweb/vaccines/who-recommendation-covid-19-vaccine-bibp.

[3] World Health Organization (2021). COVID-19 Landscape of Novel Coronavirus Candidate Vaccine Development Worldwide. https://www.who.int/publications/m/item/draft-landscape-of-covid-19-candidate-vaccines.

[4] Xia S., Zhang Y., Wang Y., Wang H., Yang Y., Gao G.F., Tan W., Wu G., Xu M., Lou Z. (2021). Safety and immunogenicity of an inactivated SARS-CoV-2 vaccine, BBIBP-CorV: A randomised, double-blind, placebo-controlled, phase 1/2 trial. Lancet Infect. Dis..

[5] Elgendy M.O., El-Gendy A.O., Mahmoud S., Mohammed T.Y., Abdelrahim M., Sayed A.M. (2022). Side Effects and Efficacy of COVID-19 Vaccines among the Egyptian Population. Vaccines.

[6] Al Kaabi N., Zhang Y., Xia S., Yang Y., Al Qahtani M.M., Abdulrazzaq N., Al Nusair M., Hassany M., Jawad J.S., Abdalla J. (2021). Effect of 2 Inactivated SARS-CoV-2 Vaccines on Symptomatic COVID-19 Infection in Adults: A Randomized Clinical Trial. JAMA.

[7] Xia S., Zhang Y., Wang Y., Wang H., Yang Y., Gao G.F., Tan W., Wu G., Xu M., Lou Z. (2022). Safety and immunogenicity of an inactivated COVID-19 vaccine, BBIBP-CorV, in people younger than 18 years: A randomized, double-blind, controlled, phase 1/2 trial. Lancet Infect. Dis..

[8] Ai J., Zhang H., Zhang Y., Lin K., Zhang Y., Wu J., Wan Y., Huang Y., Song J., Fu Z. (2022). Omicron variant showed lower neutralizing sensitivity than other SARS-CoV-2 variants to immune sera elicited by vaccines after boost. Emerg. Microbes Infect..

[9] Wang X., Zhao X., Song J., Wu J., Zhu Y., Li M., Cui Y., Chen Y., Yang L., Liu J. (2022). Homologous or heterologous booster of inactivated vaccine reduces SARS-CoV-2 Omicron variant escape from neutralizing antibodies. Emerg. Microbes Infect..

[10] Moghnieh R., Mekdashi R., El-Hassan S., Abdallah D., Jisr T., Bader M., Jizi I., Sayegh M.H., Bizri A.R. (2021). Immunogenicity and reactogenicity of BNT162b2 booster in BBIBP-CorV-vaccinated individuals compared with homologous BNT162b2 vaccination: Results of a pilot prospective cohort study from Lebanon. Vaccine.

[11] Ai J., Zhang Y., Zhang H., Zhang Q., Fu Z., Lin K., Song J., Zhao Y., Fan M., Wang H. (2022). Safety and immunogenicity of a third-dose homologous BBIBP-CorV boosting vaccination: Interim results from a prospective open-label study. Emerg. Microbes Infect..

[12] Zhang Y., Yang Y., Qiao N., Wang X., Ding L., Zhu X., Liang Y., Han Z., Liu F., Zhang X. (2022). Early assessment of the safety and immunogenicity of a third dose (booster) of COVID-19 immunization in Chinese adults. Front. Med..

[13] Etemadifar M., Abhari A.P., Nouri H., Sigari A.A., Piran Daliyeh S.M., Maracy M.R., Salari M., Maleki S., Sedaghat N. (2022). Self-Reported safety of the BBIBP-CorV (Sinopharm) COVID-19 vaccine among Iranian people with multiple sclerosis. Hum. Vaccines Immunother..

[14] Ariamanesh M., Porouhan P., PeyroShabany B., Fazilat-Panah D., Dehghani M., Nabavifard M., Hatami F., Fereidouni M., Welsh J.S., Javadinia S.A. (2022). Immunogenicity and Safety of the Inactivated SARS-CoV-2 Vaccine (BBIBP-CorV) in Patients with Malignancy. Cancer Investig..

[15] Assawakosri S., Kanokudom S., Suntronwong N., Auphimai C., Nilyanimit P., Vichaiwattana P., Thongmee T., Duangchinda T., Chantima W., Pakchotanon P. (2022). Neutralizing Activities against the Omicron Variant after a Heterologous Booster in Healthy Adults Receiving Two Doses of CoronaVac Vaccination. J. Infect. Dis..

[16] World Health Organization (2021). Evidence Assessment: Sinopharm/BBIBP COVID-19 Vaccine. https://cdn.who.int/media/docs/default-source/immunization/sage/2021/april/2_sage29apr2021_critical-evidence_sinopharm.pdf.

[17] Jeewandara C., Aberathna I., Pushpakumara P., Kamaladasa A., Guruge D., Wijesinghe A., Gunasekera B., Tanussiya S., Kuruppu H., Ranasinghe T. (2021). Persistence of antibody and T cell responses to the Sinopharm/BBIBP-CorV vaccine in Sri Lankan individuals. medRxiv.

[18] Badano M.N., Sabbione F., Keitelman I., Pereson M., Aloisi N., Colado A., Ramos M.V., Wilczyñski J.M.O., Pozner R.G., Castillo L. (2022). Humoral response to the BBIBP-CorV vaccine over time in healthcare workers with or without exposure to SARS-CoV-2. Mol. Immunol..

[19] Yu X., Wei D., Xu W., Liu C., Guo W., Li X., Tan W., Liu L., Zhang X., Qu J. (2022). Neutralizing activity of BBIBP-CorV vaccine-elicited sera against Beta, Delta and other SARS-CoV-2 variants of concern. Nat. Commun..

[20] AlHosani F.I., Stanciole A.E., Aden B., Timoshkin A., Najim O., Zaher W.A., AlDhaheri F.A., Al Mazrouie S., Rizvi T.A., Mustafa F. (2022). Impact of the Sinopharm's BBIBP-CorV vaccine in preventing hospital admissions and death in infected vaccinees: Results from a retrospective study in the emirate of Abu Dhabi, United Arab Emirates (UAE). Vaccine.

[21] Lijeskić O., Klun I., Stamenov Djaković M., Gligorić N., Štajner T., Srbljanović J., Djurković-Djaković O. (2021). Prospective Cohort Study of the Kinetics of Specific Antibodies to SARS-CoV-2 Infection and to Four SARS-CoV-2 Vaccines Available in Serbia, and Vaccine Effectiveness: A 3-Month Interim Report. Vaccines.

[22] Silva-Valencia J., Soto-Becerra P., Escobar-Agreda S., Fernández-Navarro M., Moscoso-Porras M., Solari L., Mayta-Tristán P. Effectiveness of the BBIPB-CorV Vaccine in Preventing Infection and Death in Health Care Workers in Peru 2021. https://papers.ssrn.com/sol3/papers.cfm?abstract_id=3922632.

[23] Li X.N., Huang Y., Wang W., Jing Q.L., Zhang C.H., Qin P.Z., Guan W.-J., Gan L., Li Y.-L., Liu W.-H. (2021). Effectiveness of inactivated SARS-CoV-2 vaccines against the Delta variant infection in Guangzhou: A test-negative case-control real-world study. Emerg. Microbes Infect..

[24] Wu D., Zhang Y., Tang L., Wang F., Ye Y., Ma C., Zheng H., Yu W., Cao L., Song Y. (2022). Effectiveness of Inactivated COVID-19 Vaccines Against Symptomatic, Pneumonia, and Severe Disease Caused by the Delta Variant: Real World Study and Evidence—China, 2021. China CDC Wkly..

[25] Mousa M., Albreiki M., Alshehhi F., AlShamsiv S., Al Marzouqi N., Alawadi T., Alrand H., Alsafar H., Fikri A. (2022). Similar effectiveness of the inactivated vaccine BBIBP-CorV (Sinopharm) and the mRNA vaccine BNT162b2 (Pfizer-BioNTech) against COVID-19 related hospitalizations during the Delta outbreak in the United Arab Emirates. J. Travel Med..

[26] Rearte A., Castelli J.M., Rearte R., Fuentes N., Pennini V., Pesce M., Barbeira P.B., Iummato L.E., Laurora M., Bartolomeu M.L. (2022). Effectiveness of rAd26-rAd5, ChAdOx1 nCoV-19, and BBIBP-CorV vaccines for risk of infection with SARS-CoV-2 and death due to COVID-19 in people older than 60 years in Argentina: A test-negative, case-control, and retrospective longitudinal study. Lancet.

[27] Macchia A., Ferrante D., Angeleri P., Biscayart C., Mariani J., Esteban S., Tablado M.R., de Quirós F.G.B. (2021). Evaluation of a COVID-19 Vaccine Campaign and SARS-CoV-2 Infection and Mortality Among Adults Aged 60 Years And Older in a Middle-Income Country. JAMA Netw. Open.

